# Coincidence of cutaneous blastic plasmacytoid dendritic cell neoplasm and myelodysplastic syndrome derived from clonal hematopoiesis

**DOI:** 10.1038/s41408-023-00893-9

**Published:** 2023-08-09

**Authors:** Tomohiko Yamada, Nobuhiro Hiramoto, Takuto Mori, Daisuke Yamashita, Yukimasa Tai, Ryusuke Yamamoto, Masashi Nishikubo, Hayato Maruoka, Kana Sakamoto, Kengo Takeuchi, Yasuhito Nannya, Seishi Ogawa, Takayuki Ishikawa

**Affiliations:** 1 Department of Hematology, Kobe City Medical Center General Hospital, Kobe, Japan; 2 Department of Pathology and Tumor Biology, Kyoto University, Kyoto, Japan; 3 Department of Pathology, Kobe City Medical Center General Hospital, Kobe, Japan; 4 Department of Dermatology, Kobe City Medical Center General Hospital, Kobe, Japan; 5 Department of Clinical Laboratory, Kobe City Medical Center General Hospital, Kobe, Japan; 6 Division of Pathology, Cancer Institute, Japanese Foundation for Cancer Research, Tokyo, Japan; 7 Pathology Project for Molecular Targets, Cancer Institute, Japanese Foundation for Cancer Research, Tokyo, Japan; 8 Department of Pathology, Cancer Institute Hospital, Japanese Foundation for Cancer Research, Tokyo, Japan; 9 Division of Hematopoietic Disease Control, Institute of Medical Science, The University of Tokyo, Tokyo, Japan; 10 Institute for the Advanced Study of Human Biology (WPI-ASHBi), Kyoto University, Kyoto, Japan

**Keywords:** Cancer epigenetics, Cancer epigenetics

Dear Editor,

Blastic plasmacytoid dendritic cell neoplasm (BPDCN) is a rare neoplasm derived from plasmacytoid dendritic cells of myeloid origin [1]. Karyotypic abnormalities are present in 55–65% of patients, with complex karyotypes and losses outweighing gains observed in up to 75% of cases [2]. Recurrent mutations in genes, including *TET2*, *ASXL1*, *ZRSR2*, *SRSF2*, *IDH2*, *EZH2*, and *SF3B1*, predominantly affecting DNA methylation or chromatin remodeling pathways similar to other myeloid neoplasms, have been identified through next-generation sequencing (NGS) [3, 4]. In addition, copy number variants (CNVs) involving *CDKN2A/2B*, *CDKN1B*, *IKZF1*, *ETV6*, and *RB1* have also been documented [2].

Approximately 10–20% of BPDCN patients have been reported to occur in the setting of prior or concomitant hematologic malignancies (PCHM), including myelodysplastic syndrome (MDS) and chronic myelomonocytic leukemia (CMML) [5]. Several case reports separately performed NGS on BPDCN and CMML samples from patients who developed BPDCN after a preceded history of CMML. These reports demonstrated a shared genetic clonal origin and distinct genetic clonal evolution of BPDCN and CMML [6–8]. In contrast, case reports of the shared clonal origin of BPDCN and MDS are rare [9].

This report describes a patient diagnosed with BPDCN limited to the skin and MDS with increased blasts 1 (MDS-IB1) in the bone marrow (BM). Both samples were analyzed using targeted NGS and copy number analyses (CNA). Based on the presence of common gene mutations, we confirmed a common clonal origin of both conditions. Furthermore, BPDCN and MDS evolved from a shared clonal origin through the acquisition of multiple CNVs and additional gene mutations, respectively.

**Fig. 1:**
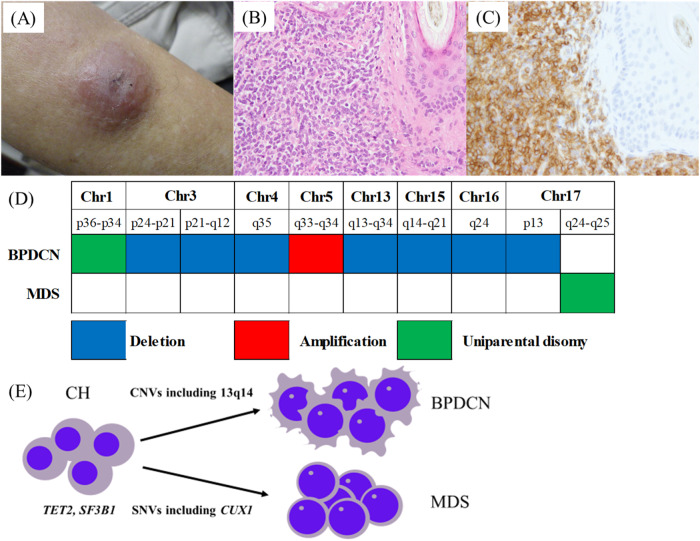
Histopathology of BPDCN, copy number variants in BPDCN and MDS, and schematic model illustrating clonal evolution of BPDCN and MDS from clonal hematopoiesis. **A** The skin lesion of the patient. **B** Histopathology of the skin leision. Hematoxylin and eosin staining (×400) showed a diffuse infiltration of blastic cells extending from the hypodermis to the subcutaneous adipose tissue. **C** Immunohistochemical staining (×400) showed the blastic cells positive for CD123. **D** Copy number variants (CNVs) in BPDCN and MDS. **E** Schematic model illustrating clonal evolution of BPDCN and MDS from clonal hematopoiesis (CH) by acquisition of multiple CNVs and additional single nucleotide variants (SNVs), respectively.

An 80-year-old male patient was referred to our hospital with a progressive cutaneous mass on the right forearm, which had been present for 3 months. The patient had no previous medical history or family history of malignancy. During physical examination, a reddish dome-shaped mass measuring 4 cm in diameter was observed on his right forearm. (Fig. 1A). His spleen and liver were not palpable. Laboratory results showed a low hemoglobin level of 10.0 g/dl, with a high mean corpuscular volume of 113.0 fl. White blood cell and platelet counts were within normal range, as were the result of liver and renal function tests. Positron emission tomography-computed tomography showed no evidence of F-18 fluorodeoxyglucose uptake except in the right forearm. The skin biopsy specimen showed diffuse infiltration of blastic cells extending from the hypodermis to the subcutaneous adipose tissue. (Fig. 1B, C). Immunohistochemical staining confirmed the diagnosis of BPDCN as the blastic cells were positive for CD4, CD56, CD123, and TCF4, and negative for CD3, CD19, lysozyme, and myeloperoxidase. Multicolor flow cytometry analysis indicated positive for CD4, CD56, CD123, BDCA-2, cytoplasmic TCL-1, and NG2 and negative for cytoplasmic CD3, cytoplasmic MPO, CD19, and CD64. In contrast, BM aspirate smears showed the presence of 5.2% myeloblasts with a dysplastic morphology, including ring sideroblasts and small megakaryocytes, consistent with MDS-IB1. In addition, multicolor flow cytometry analysis of the BM sample showed no involvement of BPDCN (<0.05% of total nucleated cells were CD4+, CD45+ [dim], CD56+, CD123+, and NG2+). The patient was treated with local radiotherapy on his right forearm and achieved complete remission of BPDCN. However, six months later, his BPDCN relapsed on the whole-body skin, while the BM showed MDS-IB1 without evidence of BPDCN. He was then treated with venetoclax plus azacytidine. After 18 cycles of treatment, the patient remains alive and in complete remission of BPDCN, with no progression of MDS-IB1.

Genetic studies were performed to analyze the clonal relationship between the BPDCN and the MDS at diagnosis, in accordance with the principles of the Declaration of Helsinki and with approval from the Ethics Committee of the Graduate School of Medicine, Kyoto University, and Kobe City Medical Center General Hospital. Furthermore, written informed consent was obtained from the patient. The methods of genetic studies are described in the Supplemental Methods.

DNA extracted from the skin (BPDCN) and BM (MDS) samples were analyzed using targeted NGS for 377 recurrently mutated genes in myeloid neoplasms. The targeted sequencing approach successfully identified common mutations, namely *TET2* (c.C5038T, p.Q1680X) and *SF3B1* (c.A2098G, p.K700E) in both skin and BM samples. In addition, targeted NGS identified *CUX1* (c.C952T, p.Q323X), *TET2* (c.G3501T, p.R1167S), and *ZRSR2* (c.T227A, p.L76X) mutations in the BM, whereas no somatic mutations were detected in the skin. The variant allele frequencies (VAFs) of the *TET2* (c.C5038T, p.Q1680X) and *SF3B1* (c.A2098G, p.K700E) mutations were 42% and 46% in the skin, and 42% and 44% in the BM, respectively (Table 1).

**Table 1:** Results of targeted sequencing in BPDCN and MDS.

Gene	Mutation	Nucleotide	Amino acid	BPDCN	MDS
	type	change	change	VAF	VAF
Common for BPDCN and MDS					
*SF3B1*	Missense	c.A2098G	p.K700E	0.46	0.44
*TET2*	Nonsense	c.C5038T	p.Q1680X	0.42	0.42
Specific for BPDCN					
Negative	N.A	N.A	N.A	N.A	N.A
Specific for MDS					
*TET2*	Missense	c.G3501T	p.R1167S	0	0.03
*CUX1*	Nonsense	c.C952T	p.Q318X	0	0.27
*ZRSR2*	Nonsense	c.T227A	p.L76X	0	0.03

*BPDCN* blastic plasmacytoid dendritic cell neoplasm, *MDS* myelodysplastic syndrome, *N.A.* not applicable.

Subsequently, we performed CNA analysis and observed multiple CNVs, including loss of 13q14 (Supplementary Figure 1; Supplementary Table 1), in the skin, while CNVs were not identified in the BM (Fig. 1D).

In summary, these genetic studies showed that (1) both BPDCN and MDS shared common *TET2* (c.C5038T, p.Q1680X) and *SF3B1* mutations; (2) the VAFs of the common mutations were higher than those of the other mutations; and (3) *CUX1*, *TET2* (c.G3501T, p.R1167S), and *ZRSR2* mutations were exclusively present in MDS, while multiple CNAs, including loss of 13q14, were specific to BPDCN (Fig. 1E).

Khanlari et al. evaluated mutations in BM hematopoietic and BPDCN cells and reported that two-thirds of BPDCN patients exhibited BM clonal hematopoiesis (CH), and half of the patients with paired samples demonstrated a clonal relationship between BM CH and BPDCN [9]. In addition, Li et al. performed genetic analysis in a case of BPDCN following clonal cytopenia of unknown significance (CCUS) and found the common *TET2* and *ZRSR2* mutations [10]. In our patient, the common *TET2* (c.C5038T, p.Q1680X) and *SF3B1* mutations overlapped with those of CH of indeterminate potential (CHIP). The higher VAFs of the common mutations compared to the other mutations suggest that the genetic events occurred in the early phase and could have originated from CHIP.

To date, several case reports have documented the shared clonal origin of BPDCN and CMML [6–8]. However, case reports on the common clonal origins of BPDCN and MDS are rare. In a targeted sequencing study by Khanlari et al. [9], involving a large cohort of 51 BPDCN patients, three cases (patient ID; 17, 19, and 48) exhibited a shared clonal origin of BPDCN and MDS, all of which had the *TET2* mutation. One of these cases (patient ID 17) also shared the *SF3B1* mutation, similar to ours. Additionally, two of the three BPDCN samples (patient ID: 17 and 19) showed multiple gene mutations, including *NRAS*, while the remaining sample (patient ID: 48) did not acquire any additional gene mutations. However, according to conventional karyotyping, the BPDCN sample has a complex karyotype, whereas the MDS sample has a diploid karyotype. In our patient, the BPDCN sample did not acquire additional gene mutations other than the common gene mutations and showed a diploid karyotype by conventional karyotyping. Nevertheless, CNA demonstrated that the BPDCN sample had acquired multiple CNVs, including a monoallelic loss of 13q14, which was not observed in the MDS sample. Therefore, in addition to targeted sequencing, considering CNA as an essential genetic study is crucial for detecting recurrent cytogenetic abnormalities in BPDCN.

Several studies investigating CNA in BPDCN have frequently identified complex chromosomal abnormalities with a predominance of losses over gains. Recurrent losses include tumor suppressor genes, especially those controlling the G1/S cell cycle transition: *CDKN2A/2B* (9q21), *CDKN1B* (12p13), and *RB1* (13q14) [2]. Notably, Patnaik et al. performed whole-exome sequencing in a case with BPDCN following CMML, and demonstrated that biallelic loss of *RBI* was exclusively detected in BPDCN, suggesting its contribution to the phenotypic transformation of BPDCN from CMML [7]. However, it remains unclear whether the monoallelic loss of *RB1* in our case significantly contributes to the evolution from CH to BPDCN. In addition to unknown genetic abnormalities, other factors such as epigenetic modifications, aberrant splicing, and environmental influences may be associated with genetic evolution.

In our case, flow cytometry analysis of the BM did not reveal any involvement of BPDCN. Chamoun et al. reported a case demonstrating minimal transforming disease of BPDCN in the BM before progression in a patient with MDS [11]. Additionally, El Hussein et al. described a case of polycythemia vera coexisting BPDCN in the BM [12]. Both studies utilized flow cytometry for detection, highlighting its importance in evaluating BPDCN in patients with myeloid neoplasms.

*TET2* mutations are the most common genetic abnormality in BPDCN [13]. Beird et al. demonstrated that patients with either missense or wild-type *TET2* mutations had better overall survival than patients with truncating mutations [14]. The presence of missense mutation in our patient with BPDCN may explain his prolonged overall survival. Considering that the *TET2* mutation occurs in both BPDCN and CHIP, as well as in patients with PCHM, further studies are warranted to better understand the therapeutic implications of this finding [15].

In summary, we performed targeted NGS and CNA analyses on the BPDCN and MDS samples. Our findings revealed that both originated from the same clonal origin known as CH, which subsequently evolved into BPDCN by acquiring multiple CNVs, including the loss of 13q14. Further studies are warranted to elucidate the unknown mechanisms underlying this evolutionary progression. To achieve this, we will employ multi-omics analyses, incorporating genomic, transcriptomic, and epigenomic analyses.

### Supplementary information

Supplementary information
