# Supplementary material for: Coincidence of cutaneous blastic plasmacytoid dendritic cell neoplasm and myelodysplastic syndrome derived from clonal hematopoiesis
Source: Blood Cancer J. 2023 Aug 9;13(1):119. doi: 10.1038/s41408-023-00893-9 (PMC10412548; doi:10.1038/s41408-023-00893-9)
Supplement: Supplementary file 1 — Supplementary information [file 41408_2023_893_MOESM1_ESM.docx]

**Supplementary information**

**Coincidence of cutaneous blastic plasmacytoid dendritic cell neoplasm and myelodysplastic syndrome derived from clonal hematopoiesis**

**Supplementary methods**

**Sample collection**

Genomic DNA from the skin or bone marrow of the patient was extracted using the QIAamp DNA mini kit (Qiagen) and enriched for target regions by liquid-phase hybridization using the SureSelect custom kit (Agilent Technologies^®^), according to the manufacturer’s protocol optimized for automated sample processing, as previously described.^1^

**Targeted sequencing**

The purified library was subjected to high-throughput sequencing analysis on an Illumina HiSeq 2500 or NovaSeq 6000 using the 125 bp or 150 bp paired mode. Sequencing reads were aligned to the human genome reference (hg19) using the Burrows-Wheeler Aligner (version 0.7.8) with default parameter settings. Mutation calling was performed through our established pipeline (Genomon pipeline 2.6.0, https://github.com/Genomon-Project), as previously reported.^1-4^ The significant variants that fulfilled the quality filter noted above were further assessed for oncogenicity based on an in-house curation program. The curation policy was determined individually for each gene based on previous reports and databases after excluding variants registered in public SNPs databases (the 1000 Genomes Project as of August 2014, ESP6500, Human Genome Variation Database) and call errors using EB call^5^ and an in-house blacklist of error calls.

**Copy number analysis and allelic imbalance**

We included 1,158–1,428 SNPs probes to detect copy number changes and allelic imbalances. This technique, called CNACS, was implemented using a program available at https://github.com/papaemmelab/toil_cnacs. A manual inspection of the results was conducted to identify call errors. A total copy number (TCN) of 2.22 or larger, was assumed to be CN-gains, and a TCN <1.88 was assumed to be CN-loss. A copy number-neutral LOH was defined when the B-allele frequency was <0.90, with a TCN between 1.88 and 2.22. Arm-level CNA was defined when the total length of the affected region within the arm was > 1 M bp for 17p and >3 M bp for the other arms.

**Supplementary Figure legends**

Supplementary Figure 1. Total copy number (CN) and allele specific CN states in the BPDCN and MDS samples derived with CNACS algorithm.^5^ The x-axis corresponds to genome wide chromosomal coordinates. On the upper panel, y-axis represents total CN, whereas on the lower panel y-axis represents allele specific CNs. Blue dots show total CN on coverage probes while red and green dots show major and minor CNs, respectively, on heterozygous SNP probes. Aberrant segments called by CNACS are underlined with solid lines.

**Supplementary Figure 1.**


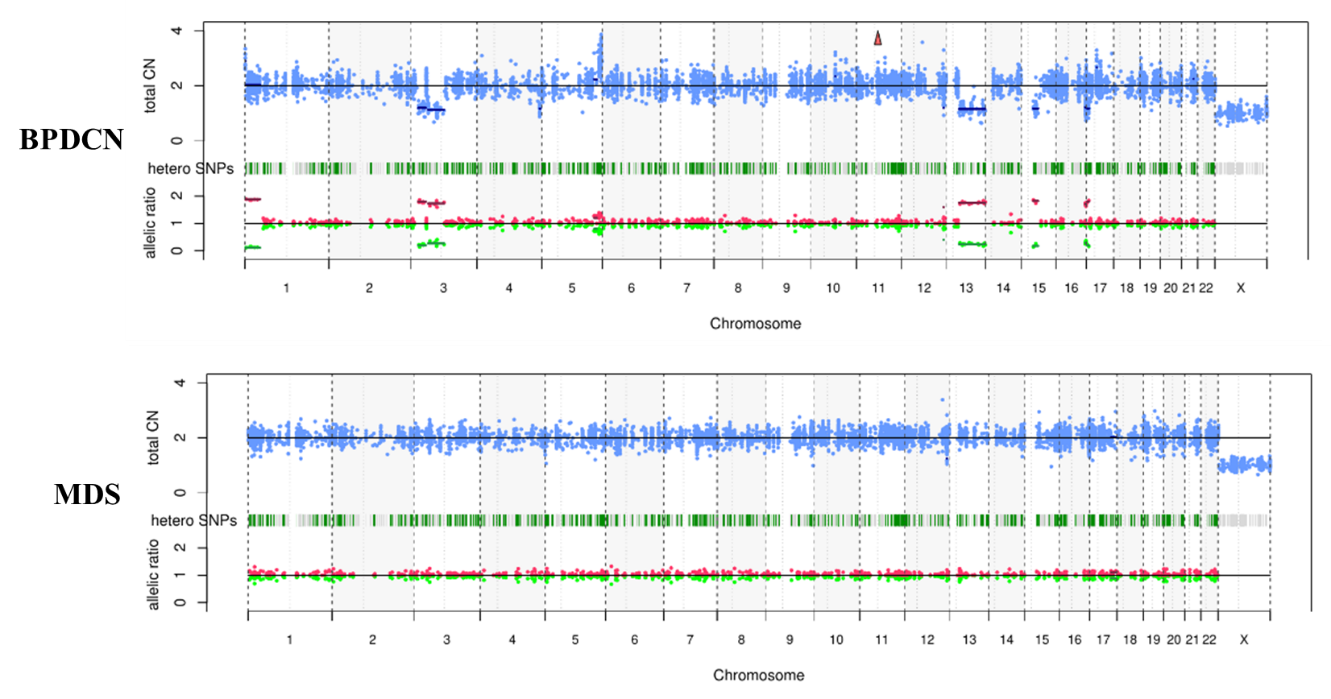


**Supplementary Table 1 Copy number analysis of BPDCN**

| **Supplementary Table 1. Copy number analysis of BPDCN** | | | | | | |
| --- | --- | --- | --- | --- | --- | --- |
| Chr | | Start | End | TCN | BAF | Genes |
| 1 | 1p36.33 | 1718702 | 1756773 | 2.90 |  | *GNB1* |
| 1 | 1p36.32-1p34.1 | 2761304 | 45032793 | 2.03 | 0.13 | *ARID1A,　CLCN6,　CSF3R,　rs3917981,　GNL2,　LIN28A,　MAD2L2,　MPL,　MTOR,　RPL11,　UBR4* |
| 3 | 3p24.3-3p21.31 | 22707679 | 45199831 | 1.19 | 0.21 | *MYD88,　RPL15,　SLC25A38* |
| 3 | 3p21.1-3q12.1 | 52436563 | 99296346 | 1.14 | 0.28 | *BAP1,　ROBO1　ROBO2* |
| 4 | 4q35.1-4q35.2 | 185309885 | 187714668 | 1.18 |  | *IRF2* |
| 5 | 5q33.3-5q34 | 155907396 | 163953041 | 2.25 |  |  |
| 10 | 10q22.1 | 73455108 | 73567563 | 2.35 |  | *CDH23* |
| 12 | 12q24.31 | 124839927 | 124848164 | 1.19 | 0.41 | *NCOR2* |
| 13 | 13q13.3-13q34 | 36453828 | 114323906 | 1.16 | 0.24 | *DIS3,　ELF1,　LIG4,　RB1* |
| 15 | 15q14-15q21.1 | 34634118 | 49220574 | 1.19 | 0.19 | *CDAN1,　MGA,　NOP10,　RAD51* |
| 16 | 16q24.1-16q24.3 | 86390216 | 88851250 | 1.22 | 0.30 | *PIEZO1,　ZFPM1* |
| 17 | 17p13.3-17p13.1 | 1225849 | 6902669 | 1.19 | 0.20 | *PRPF8* |
| 17 | 17q11.2 | 30264186 | 30325639 | 2.67 |  | *SUZ12* |
| 21 | 21q22.12 | 36289384 | 36294267 | 2.26 |  |  |
| Abbreviations: Chr; chromosome, TCN; total copy number, BAF; B-allele frequency | | | | | | |

**References**

1. Yoshida K, Sanada M, Shiraishi Y, Nowak D, Nagata Y, Yamamoto R*, et al.* Frequent pathway mutations of splicing machinery in myelodysplasia. *Nature* 2011 Sep 11; **478**(7367)**:** 64-69.

2. Haferlach T, Nagata Y, Grossmann V, Okuno Y, Bacher U, Nagae G*, et al.* Landscape of genetic lesions in 944 patients with myelodysplastic syndromes. *Leukemia* 2014 Feb; **28**(2)**:** 241-247.

3. Yoshizato T, Dumitriu B, Hosokawa K, Makishima H, Yoshida K, Townsley D*, et al.* Somatic Mutations and Clonal Hematopoiesis in Aplastic Anemia. *The New England journal of medicine* 2015 Jul 2; **373**(1)**:** 35-47.

4. Suzuki H, Aoki K, Chiba K, Sato Y, Shiozawa Y, Shiraishi Y*, et al.* Mutational landscape and clonal architecture in grade II and III gliomas. *Nature genetics* 2015 May; **47**(5)**:** 458-468.

5. Yoshizato T, Nannya Y, Atsuta Y, Shiozawa Y, Iijima-Yamashita Y, Yoshida K*, et al.* Genetic abnormalities in myelodysplasia and secondary acute myeloid leukemia: impact on outcome of stem cell transplantation. *Blood* 2017 Apr 27; **129**(17)**:** 2347-2358.
